# Letter to the editor: Severe parvovirus B19 infections in the immunocompetent population

**DOI:** 10.2807/1560-7917.ES.2024.29.29.2400438

**Published:** 2024-07-18

**Authors:** Marta Giovanetti, Francesco Branda, Fabio Scarpa, Massimo Ciccozzi, Giancarlo Ceccarelli

**Affiliations:** 1 Sciences and Technologies for Sustainable Development and One Health, Università Campus Bio-Medico di Roma, Italy; 2 Instituto Rene Rachou, Fundação Oswaldo Cruz, Minas Gerais, Brazil; 3 Climate Amplified Diseases and Epidemics (CLIMADE), Brazil, Brazil; 4 Unit of Medical Statistics and Molecular Epidemiology, Università Campus Bio-Medico di Roma, Rome, Italy; 5 Department of Biomedical Sciences, University of Sassari, Sassari, Italy; 6 Infectious Diseases Department, Azienda Ospedaliero Universitaria Policlinico Umberto I, Rome, Italy

**Keywords:** parvovirus B19, surveillance, molecular diagnosis, molecular screening, immunocompetent population, viral infection

**To the editor**: We read with great interest the report by Nordholm et al. documenting the significant increase in severe parvovirus B19 (PVB19) infections among pregnant women during the recent outbreak in Denmark [1]. Although an upsurge in PVB19 infections has been observed across northern Europe since 2023, a comprehensive epidemiological analysis remains elusive due to the lack of systematic surveillance in most countries [2]. However, the recent resurgence of PVB19 in Europe necessitates a critical reassessment of its potential clinical impact, particularly among those traditionally considered of low risk, namely immunocompetent adults and adolescents. While often perceived as a benign childhood illness with a risk of complications almost exclusively for immunocompromised individuals and during pregnancy, severe PVB19 infection is well-documented in the immunocompetent adult and adolescent population (Table) and warrants proactive vigilance, especially during epidemic periods.

**Table:** Reports of severe complications of parvovirus B19 infection in adult and adolescent patients, 2020–2024

Severe adverse complication	Number of patients	Age (years)	Sex	Co-morbidity	Supportive treatment	Survival	Reference
Decompensated liver cirrhosis in adult patients^a^							
Severe anaemia, lower resolution rate of bacterial superinfections, significantly higher 30-day mortality	10	53 (mean)	NA	Liver cirrhosis	NA	3/10	[4]
Patients living with HIV using antiretroviral treatment with stable immune virological response							
Severe anaemia	1	61	M	Chronic HIV infection	Red blood cell transfusions, glucocorticoid, IVIG	Y	[6]
Immunocompetent adults and adolescents							
Severe septic shock	1	50	F	Rheumatic valvular disease and recent elective double metal valve surgical replacement	Vasoactive pharmacologic therapy, glucocorticoid, IVIG	Y	[7]
Glomerulonephritis, respiratory failure, diffuse left ventricular hypokinesis	1	22	F	None	Artificial ventilation, glucocorticoid, IVIG	Y	[8]
Acute hepatitis, hepatosplenomegaly, polyarthritis	1	43	F	None	Ribavirin, pegylated interferon	Y	[9]
Fulminant hepatitis, acute liver failure	1	16	F	None	Therapeutic plasma exchange, candidate for liver transplantation	Y	[10]
Sudden cardiac death^b^	1	15	M	Unknown PVB19/HHV6 co-infection, hypertrophic cardiomyopathy due to mutation of *MYBPC3*	Postmortem diagnosis of viral myocarditis in combination with genetic cardiomyopathy	N	[11]
	1	60	M	Unknown PVB19/HHV6/EBV co-infection, unknown dilated cardiomyopathy due to mutation of the gene coding for desmin -*DES* gene		N	
Sudden cardiac death^c^	1	22	M	Unknown PVB19/HHV6 co-infection. Unknown carrier of a variant in the *RYR2* gene, responsible of CPVT	Postmortem diagnosis of viral myocarditis in combination with genetic cardiomyopathy	N	
Fulminant myocarditis	1	33	M	None	VA-ECMO, orthotopical heart transplant	Y	[12]
Fulminant lymphocytic myocarditis	1	18	F	Concomitant H1N1 infection	Vasoactive pharmacologic therapy, temporary left ventricle assists device (LVAD) implantation	Y	[13]
Bilateral conjunctivitis, keratitis, anterior uveitis	1	39	F	COVID-19	Glucocorticoid, dexamethasone and tropicamide eye drops	Y	[14]
Pure red cell aplasia	1	29	M	COVID-19 pneumonia and SARS-CoV-2 induced autoimmune haemolytic anaemia, obesity, asthma	Glucocorticoid, transfusion-dependency, urgent intubation	Y	[15]
GBS^d^	1	12	M	None, history of blood transfusion	Ganciclovir	NA	[16]
	1	40	M	None	IVIG, ganciclovir, immunoadsorption	Y	
Encephalitis^d^	1	14	M	Fracture reduction and fixation, history of blood transfusion	Ganciclovir	NA	
	1	30	M	Cerebral haemorrhage and removal of intracranial hematoma	Ganciclovir	NA	
	1	68	M	None	Ganciclovir	NA	
	1	50	M	Cerebral haemorrhage and removal of intracranial hematoma, history of blood transfusion	Ganciclovir	NA	
	1	66	M	Craniocerebral injury	Ganciclovir	NA	
	1	57	F	None	Ganciclovir	NA	
	1	63	M	Cerebral haemorrhage	Ganciclovir	NA	
Meningoencephalitis, GBS^d^	1	60	M	Heart valve replacement due to mitral valve prolapse, history of blood transfusion	Ganciclovir, glucocorticoid, IVIG	Y	
Meningoencephalitis, fulminant myocarditis^e^	1	13	F	None	VA – ECMO, vasoactive pharmacologic therapy, glucocorticoid, IVIG	N^f^	Personal communication Giancarlo Ceccarelli, May 2024

CPVT: catecholaminergic polymorphic ventricular tachycardia; EBV: Epstein Barr virus; F: female; GBS: Guillain–Barré syndrome; HHV6: herpesvirus type 6; IVIG: intravenous immunoglobulin; M: male; MYBPC3*:* cardiac myosin-binding protein C gene; N: no; NA: not available; PVB19: parvovirus B19; VA-ECMO: veno-arterial extracorporeal membrane oxygenation; Y: yes.

^a^ Ten (3.7%) cases in a cohort of 273 cirrhotic patients.

^b^ Suspected arrhythmic event related to PVB19 myocarditis in genetic cardiomyopathy.

^c^ Suspected opioids-induced long QT syndrome in PVB19 myocarditis.

^d^ Hospitalised patients suspected of infection with a pathogenic microorganism.

^e^ Parvovirus B19 was detected in both pericardial and cerebrospinal fluids, while other pathogens were excluded.

^f^ Death following multi-organ failure within 4 days.

Three key factors underscore the need for heightened awareness and active case finding:

Firstly, the changing immunity landscape. The recent pandemic period, characterised by lockdowns and social distancing, inadvertently suppressed PVB19 circulation. This likely led to a decline in population immunity, creating a larger pool of susceptible individuals, even among those generally considered immunocompetent [3].

Secondly, the heterogeneity within immunocompetence. The immunocompetent condition encompasses a clinically diverse spectrum of situations: in fact, while it generally implies a functioning immune system, it does not exclude the presence of underlying medical conditions, genetic predispositions or concurrent infections that could increase vulnerability to severe PVB19 infection. Co-morbidities, even those seemingly minor, can significantly alter an individual's response to the virus or interact with it to contribute to the development of severe clinical manifestations [4,5].

Lastly, the relevance of diagnostic blind spots. The misconception of PVB19 as primarily a paediatric and immunocompromised concern often leads to missed or delayed diagnoses in immunocompetent adults. Clinicians may not consider PVB19 as a differential diagnosis in immunocompetent individuals presenting with compatible symptoms, attributing them to other causes. This is particularly concerning for undiagnosed cases, as timely intervention is crucial to prevent severe complications.

In light of these considerations, a reassessment of clinical strategies during periods of heightened viral circulation seems warranted, shifting the focus from the traditional risk groups perspective, which tends to view PVB19 complications as a concern mainly for high-risk populations. During an epidemic, it seems prudent to adopt a clinical approach that systematically considers the potential presence of PVB19 infection or co-infection in all cases presenting with severe compatible symptoms, regardless of immune status. Detailed anamnesis should focus on identifying potential risk factors, including recent exposure to children, underlying medical conditions and current medications. A low threshold for PVB19 testing could mitigate the risk of clinical underestimation, especially during outbreaks, for patients with suggestive clinical presentations, even in the absence of classic risk factors.

By acknowledging the potential for severe PVB19 infection in all individuals, regardless of immune status and adopting proactive diagnostic and management strategies, the risk of serious complications could be mitigated, thereby improving patient outcomes.
